# Fucoidan from *Fucus vesiculosus* suppresses hepatitis B virus replication by enhancing extracellular signal-regulated Kinase activation

**DOI:** 10.1186/s12985-017-0848-8

**Published:** 2017-09-16

**Authors:** Huifang Li, Junru Li, Yuan Tang, Lin Lin, Zhanglian Xie, Jia Zhou, Liyun Zhang, Xiaoyong Zhang, Xiaoshan Zhao, Zhengliang Chen, Daming Zuo

**Affiliations:** 1 Department of Immunology, School of Basic Medical Sciences, Southern Medical University, Guangzhou, Guangdong 510515 China; 2 Department of Dermatology, Zhujiang Hospital, Southern Medical University, Guangzhou, Guangdong 510282 China; 3 State Key Laboratory of Organ Failure Research, Guangdong Provincial Key Laboratory of Viral Hepatitis Research, Department of Infectious Diseases, Nanfang Hospital, Southern Medical University, Guangzhou, Guangdong 510515 China; 4 School of Traditional Chinese Medicine, Southern Medical University, Guangzhou, Guangdong 510515 China; 5 Guangdong Provincial Key Laboratory of Proteomics, Southern Medical University, Guangzhou, Guangdong 510515 China

**Keywords:** Fucoidan, Hepatitis B virus, Extracellular signal-regulated kinase, Interferon, Anti-viral effect

## Abstract

**Background:**

Hepatitis B virus (HBV) infection is a serious public health problem leading to cirrhosis and hepatocellular carcinoma. As the clinical utility of current therapies is limited, the development of new therapeutic approaches for the prevention and treatment of HBV infection is imperative. Fucoidan is a natural sulfated polysaccharide that extracted from different species of brown seaweed, which was reported to exhibit various bioactivities. However, it remains unclear whether fucoidan influences HBV replication or not.

**Methods:**

The HBV-infected mouse model was established by hydrodynamic injection of HBV replicative plasmid, and the mice were treated with saline or fucoidan respectively. Besides, we also tested the inhibitory effect of fucoidan against HBV infection in HBV-transfected cell lines.

**Results:**

The result showed that fucoidan from *Fucus vesiculosus* decreased serum HBV DNA, HBsAg and HBeAg levels and hepatic HBcAg expression in HBV-infected mice. Moreover, fucoidan treatment also suppressed intracellular HBcAg expression and the secretion of the HBV DNA as well as HBsAg and HBeAg in HBV-expressing cells. Furthermore, we proved that the inhibitory activity by fucoidan was due to the activation of the extracellular signal-regulated kinase (ERK) pathway and the subsequent production of type I interferon. Using specific inhibitor of ERK pathway abrogated the fucoidan-mediated inhibition of HBV replication.

**Conclusion:**

This study highlights that fucoidan might be served as an alternative therapeutic approach for the treatment of HBV infection.

## Background

Hepatitis B virus (HBV) infection is a global public health problem. The infection can lead to acute and chronic hepatitis, which makes liver prone to develop cirrhosis and hepatocellular carcinoma [1]. Although an effective vaccine has been used for 20 years in many countries, there are still about 350 million chronic carriers of HBV worldwide, and approximately 1 million people die from HBV-related liver diseases each year [2]. The currently approved agents for the treatment of HBV infection include immunomodulatory agents, such as interferon-α (IFN-α) and pegylated IFN-α, and oral nucleoside/nucleotide analogues, like lamivudine, adefovir, telbivudine, entecavir and tenofovir [3, 4]. However, the major drawback of nucleoside/nucleotide analogues therapies is the development of drug resistance mutations with long-term treatment [3], and the disadvantages to using interferon-α are its various side effects and inconvenience of administration [4]. Therefore, exploring and developing new anti-HBV agents different from nucleoside/nucleotide analogues and interferon are urgently needed.

Fucoidan is a natural sulfated polysaccharide that extracted from different species of brown seaweed [5], which was reported to exhibit numerous biological activities, including anti-bacterial, anti-oxidant, anti-coagulant, anti-viral, anti-tumor, and anti-inflammatory effects [6–10]. These functional properties of fucoidan make it an attractive candidate for the development of biomaterials and drugs. Fucoidan isolated from *Fucus vesiculosus* is composed of 44.1% fucose, 26.3% sulfate, 31.1% ash, and a small amount of aminoglucose, which is commercially available now [5]. It has undergone extensive in vitro, in vivo as well as human clinical testing, and has also been shown to have a range of biological properties, including beneficial effects in the areas of inflammation, immune modulation, and enzyme inhibition [11]. There are several literatures concerning the anti-viral bioactivity of fucoidan isolated from *Fucus vesiculosus* [11–13]. Beress et al.*,* described an anti-human immunodeficiency virus (HIV) activity of some fractions of self-produced *Fucus vesiculosus* fucoidan [12]. Besides, fucoidan displayed antiviral activities against influenza A infection by different subtypes [14, 15]. A recent clinical study showed that treatment with fucoidan suppressed the expression of hepatitis C virus (HCV) replicon, accompanied by limited serum alanine aminotransferase levels, in HCV-infected patients [16]. However, it remains to be studied whether and how fucoidan inhibits HBV replication in vivo and in vitro.

In this study, we determined that fucoidan isolated from *Fucus vesiculosus* effectively inhibited HBV replication in an HBV replication mouse model in vivo and in HepG2.2.15 cells in vitro. Furthermore, we provided the evidence that fucoidan activated mitogen-activated protein kinases (MAPKs) extracellular signal-regulated kinase 1/2 (ERK1/2) pathway and subsequently promoted the expression of IFN-α, leading the decreased production of HBV DNA and proteins. These data suggest the possibility of using fucoidan as an alternative therapeutic strategy for HBV infection.

## Methods

### Animal

C57BL/6 mice (male, 6–8 weeks old) were purchased from the Laboratory Animal Center of Southern Medical University (Guangzhou, China). The HBV infection mouse model was established as described previously [17]. Briefly, 10 μg of pHBV1.3 plasmid, carrying a terminally redundant (1.3-fold) replication-competent HBV genome, was injected into the tail vein of mice with in 5 s in a volume of PBS equivalent to 8% of the mouse body weight. For fucoidan treatment, HBV-infected mice were intraperitoneally injected with 100 mg fucoidan at 0, 1, 3, 5, and 7 days post-infection. The levels of HBV DNA, HBsAg and HBeAg in the serum and the expression of HBcAg in liver tissues of mice were detected at the indicated time point.

### Reagents and antibodies

Fucoidan from *Fucus vesiculosus* was purchased from Sigma (St. Louis, Mo, USA.). Human IFN-α ELISA kit and mouse IFN-α ELISA kit were purchased from Cloud-Clone Corp (Wuhan, China). The primary antibodies were purchased from Cell Signaling Technology (Danvers, MA, USA), including the antibodies against phospho-ERK1/2, ERK1/2, phpspho-p38, p38, phospho-JNK, JNK, phospho-IRF3, IRF3 and phospho-IRF7, IRF7, and GAPDH. The antibodies against HBsAg and HBcAg were obtained from Thermo Fisher (MS-314, RB-1413, Thermo Fisher, IL, U.S.A.). ERK inhibitor U0126 was from Cell Signaling Technology.

### Cell culture and transfection

The HBV-producing cell line HepG2.2.15 were maintained in RPMI 1640 medium supplemented with 10% fetal bovine serum and 400 μg/mL of G418, and incubated at 37 °C in an atmosphere of 5% CO_2_. HepG2 cells were seeded in 6-well plates at a density of 6 × 10^5^ cells per well and were transfected with 3 μg of plasmid pHBV1.3 with PEI (Polysciences, Warrington, PA, USA) following the manufacturer’s instruction.

### Cytotoxicity assay

HepG2.2.15 cells (1 × 10^4^ cells/well) were cultured in 96-well plates and incubated with varying concentrations of fucoidan for 24 or 48 h. Cytotoxic effect of fucoidan on HepG2.2.15 cells was measured via CCK-8 assays (Dojindo Laboratories, Kumamoto, Japan) following the manufacturer’s instruction. The absorbance at 450 nm was measured using a microplate reader.

### Determination of HBsAg and HBeAg

HepG2.2.15 cells were seeded in 6-well plates at a density of 1 × 10^6^ cells per well and cultured for 12 h, then treated with indicated concentrations of fucoidan for another 24 h. The levels of HBsAg and HBeAg in the supernatants were determined using ELISA kits (RongSheng Bioengineering, Shanghai, China) according to the manufacturer’s instruction. The absorbance at 450 nm was measured by a microplate reader.

### Western blot analysis

Whole cell protein lysates were extracted in the cell lysis buffer (Beyotime Biotechnology, Shanghai, China). Equal amounts of protein extracts were separated on SDS-polyacrylamide gels and then transferred onto polyvinylidene fluoride (PVDF) membranes (Millipore, Billerica, MA, USA). After blocking with bovine serum albumin (BSA, 5%) at room temperature for at least 1 h, membranes were incubated overnight at 4 °C with primary antibodies, followed by incubation with the horseradish peroxidase-conjugated secondary antibody for 1 h at room temperature. Finally, the membranes were washed three times, and detection of the target protein was conducted with enhanced chemiluminescence (Thermo Fisher).

### Southern blot analysis

The isolation of HBV DNA replication intermediates were performed as previously described [18]. Samples were electrophoresed into agarose gels and blotted onto Hybond-XL membranes (GE Healthcare, Marlborough, MA, USA). Then, the membranes were probed with a DIG-labeled specific full-length HBV riboprobe, generated using the PCR DIG Probe Synthesis Kit (Roche, Basel, Switzerland), and visualized with CDP-Star using an ImageQuant LAS 2000 mini system (GE Healthcare).

### HBV DNA isolation and real-time PCR

Intracellular nucleocapsid-associated viral DNA was extracted from HepG2.2.15 cells. SYBR Green quantitative RT-PCR was performed to determine the gene expression level using a 7900HT fast real-time PCR system (Applied Biosystems, San Francisco, CA, USA), according to the protocols provided with the SYBR Premix EX Taq (TaKaRa). The primers used to amplify were: HBV DNA (forward 5′-ACC AAT CGC CAG TCA GGA AG-3′ and reverse 5′-ACC AGC AGG GAA ATA CAG GC-3′); GAPDH (forward 5′-CAT CAC TGC CAC CCA GAA GAC TG-3′ and reverse 5′-ATG CCA GTG AGC TTC CCG TTC AG-3′). The levels of target gene were normalized with respect to GAPDH gene expression.

### Northern blot analysis

Total RNA was isolated from HepG2.2.15 cells using Trizol reagent (Invitrogen). For Northern blotting, 30 μg total RNA was electrophoresed in a 1% formaldehyde agarose gel containing 5% formaldehyde and blotted onto Hybond-XL membranes (GE Healthcare, Marlborough, MA, USA). Then, the membrane was probed with a DIG-labeled specific full-length HBV riboprobe, generated using the PCR DIG Probe Synthesis Kit (Roche, Basel, Switzerland), and visualized with CDP-Star using an ImageQuant LAS 2000 mini system (GE Healthcare). Finally, the blot was stripped and rehybridized with a DIG-labeled GAPDH probe for normalization.

### Statistical analysis

The experimental data were evaluated by calculating the mean ± SD. One-way ANOVA was used for comparisons among multiple groups. Differences between two groups within experiments were analyzed by Student’s *t*-test. All the experiments were repeated at least three times independently. Values of *p* < 0.05 were considered statistically significant.

## Results

### Inhibitory activity of fucoidan on HBV replication in vivo

**Fig. 1:**
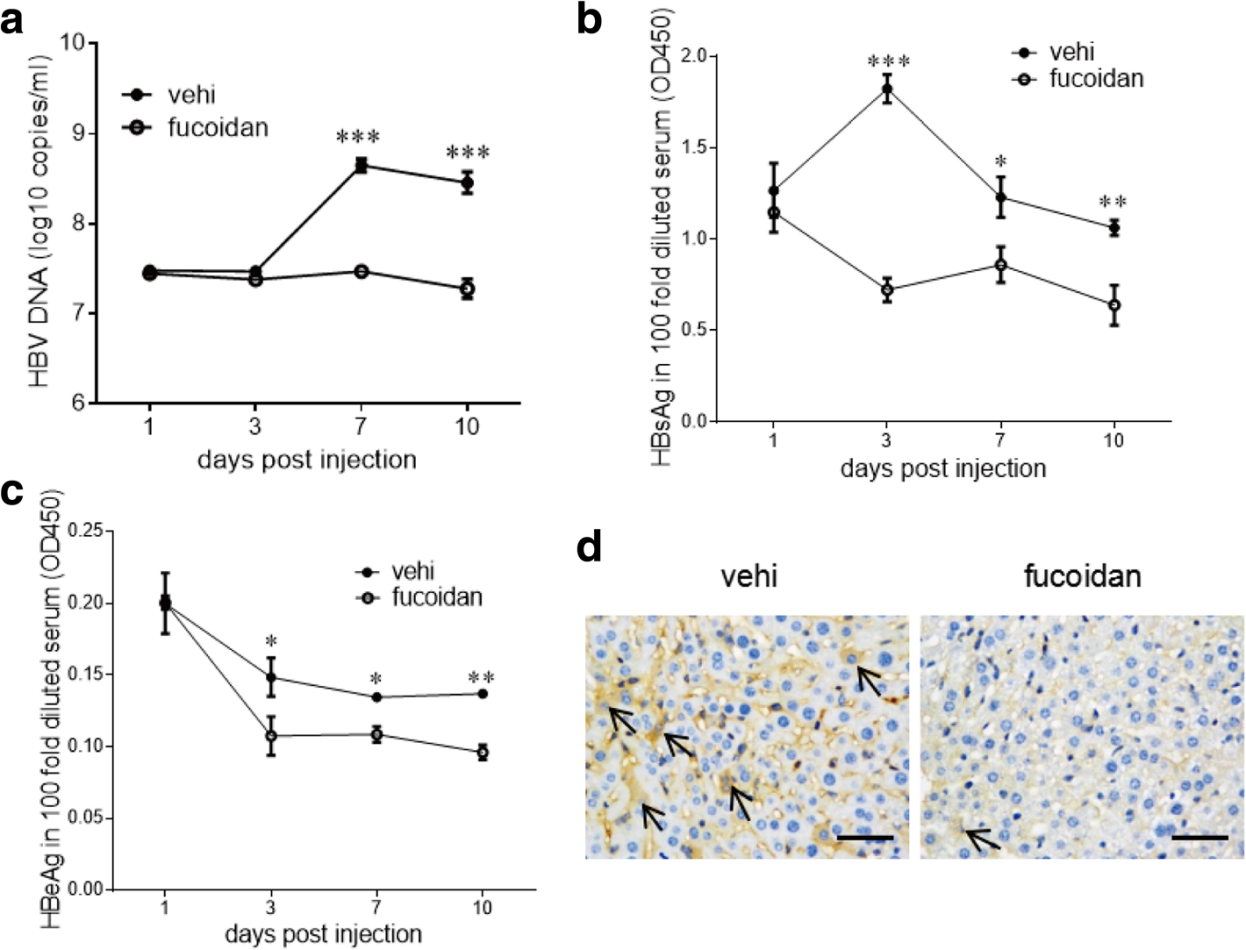
Fucoidan exhibits anti-HBV activity in HBV-infected mice. C57BL/6 mice were hydrodynamically injected with 10 μg of pHBV1.3 plasmids through the tail vein. The mice were intraperitoneally administrated with 100 mg fucoidan at 0, 1, 3, 5, and 7 days post-infection. **a** The HBV DNA replicative intermediates in the serum were evaluated by quantitative PCR. **b**, **c** The levels of HBsAg (**b**) and HBeAg (**c**) in the sera were measured by ELISA. * *p* < 0.05, ** *p* < 0.01, *** *p* < 0.001. **d** The expression of HBcAg in the mice liver tissue was detected by immunohistochemistry at day 10 post-infection. Arrows indicate HBcAg-positive cells. Scale bar = 100 μm. Data are representative of three independent experiments with similar results

We first investigated whether fucoidan can inhibit HBV replication in vivo. C57BL/6 mice were hydrodynamic-injected with 10 μg of pHBV1.3 plasmid through the tail vein. Meanwhile, the mice were intraperitoneally administered with 100 mg of fucoidan at 0, 1, 3, 5, and 7 days post-infection. Mouse serum was collected at indicated time points after the injection. Serum HBV DNA was analyzed by using real-time PCR, and HBsAg/HBeAg levels were analyzed by using ELISA. As shown in Fig. 1a, serum HBV DNA level was significantly reduced after injection of fucoidan. Moreover, fucoidan administration also suppressed HBsAg and HBeAg secretion (Fig. 1b and c). Immunohistochemical analysis showed that fucoidan suppressed the HBcAg expression in the liver tissues from HBV-infected mice (Fig. 1d). These results indicated that fucoidan could inhibit HBV replication in vivo.

### Fucoidan inhibited the HBV replication in vitro

**Fig. 2:**
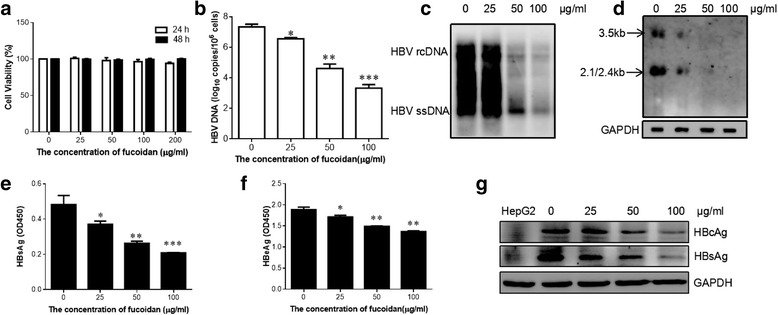
Fucoidan suppresses HBV replication in the HepG2.2.15 cell line. **a** Cells were incubated with varying concentrations of fucoidan for 24 or 48 h. Cytotoxic effect of fucoidan on HepG2.2.15 cells was measured via CCK-8 assays. **b**-**g** HepG2.2.15 cells were treated with the indicated concentrations of fucoidan for 24 h. **b**, **c** The HBV DNA replicative intermediates were determined by quantitative PCR (**b**) and Southern blotting (**c**). **d** Northern blotting was performed to measure the intracellular HBV RNA levels. **e**, **f** The levels of HBsAg (**e**) and HBeAg (**f**) in the cell culture medium were analyzed via ELISA. **g** The expressions of HBsAg and HBcAg in HepG2.2.15 cells were examined by western blotting analysis. HepG2 cells was served as a negative control. * *p* < 0.05, ** *p* < 0.01, *** *p* < 0.001, compared to the group without fucoidan treatment. All the experiments were repeated at least three times independently

Next, we evaluated the effect of fucoidan on HBV replication in vitro. CCK8 assay showed that fucoidan was not cytotoxic at concentrations lower than 200 μg/ml on HepG2.2.15 cells (Fig. 2a). Real-time PCR revealed that fucoidan treatment reduced the levels of HBV DNA replicative intermediates in a dose-dependent manner (Fig. 2b). Additionally, the inhibitory effect of fucoidan on HBV DNA replicative intermediates was confirmed by southern blotting analysis (Fig. 2c). To explore whether the observed reduction of HBV DNA replication in HepG2.2.15 cells by fucoidan was due to its impact on HBV RNA synthesis. Northern blotting was performed to measure the intracellular HBV RNA levels after fucoidan treatment. The results showed that treatment with different concentrations of fucoidan induced a dose-dependent decrease in the levels of HBV RNA in HepG2.2.15 cells (Fig.2d). Secretion of HBsAg and HBeAg in the culture supernatants was also significantly decreased in the fucoidan-treated cells compared to the control cells (Fig. 2e and f). Western blotting showed that fucoidan markedly limited the expression of HBsAg and HBcAg in HepG2.2.15 cells (Fig. 2g).

**Fig. 3:**
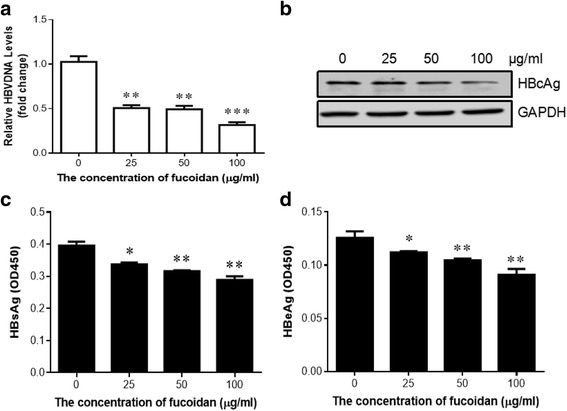
Fucoidan reduces HBV replication in hepatocyte-derived cells. HepG2 cells were transfected with pHBV1.3 as mentioned. 24 h later, cells were treated with indicated concentrations for another 24 h. **a** The level of HBV DNA in the cell lysate was detected using quantitative PCR. **b** The expression of HBcAg in the cells was determined by western blot. **c**, **d** The productions of HBsAg (**c**) and HBeAg (**d**) in the culture supernatants were evaluated using ELISA. * *p* < 0.05, ** *p* < 0.01, compared to the group without fucoidan treatment. All the experiments were repeated at least three times independently

To further confirm the inhibitory effect of fucoidan on HBV replication, HepG2 cells were transiently transfected with pHBV1.3 plasmid and subsequently incubated with indicated concentrations of fucoidan. Quantitative PCR result showed that fucoidan suppressed the level of HBV DNA replicative intermediates in a dose-dependent manner (Fig. 3a). Western blotting showed that fucoidan treatment markedly limited HBV core protein expression in HBV-infected HepG2 cells (Fig. 3b). ELISA analysis revealed that fucoidan decreased the secretion of HBsAg and HBeAg in the culture medium (Fig. 3c and d). Taken together, these data suggested that fucoidan repressed HBV replication in vitro.

### Fucoidan enhanced the activation of ERK pathway and facilitated the production of type I interferon

**Fig. 4:**
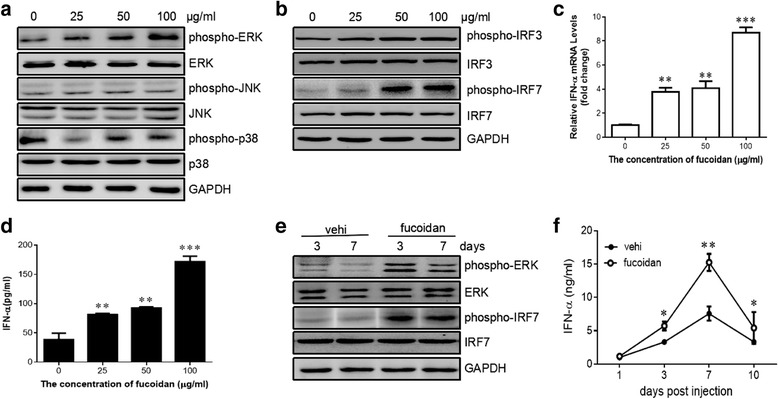
Fucoidan promotes the activation of ERK pathway and enhances the production of type I interferon in vitro and in vivo. **a**-**d** HepG2.2.15 cells were incubated with indicated concentrations of fucoidan for 24 h. **a** Phospho-ERK, total ERK, phospho-JNK, total JNK, phospho-p38, and total p38 were determined by western blotting. **b** Phospho-IRF3, total IRF3, phospho-IRF7, and total IRF7 were examined by western blotting. **c** The production of IFN-α in the culture supernatant was evaluated by ELISA analysis. **d** The mRNA level of IFN-α in cells was determined by quantitative RT-PCR. **e**, **f** C57BL/6 mice were hydrodynamically injected with 10 μg of pHBV1.3 plasmids through the tail vein. The mice were intraperitoneally administrated with 100 mg fucoidan at 0, 1, 3, 5, and 7 days post-infection. **e** Phospho-ERK, total ERK, phospho-IRF7, and total IRF7 in the liver tissue of infected mice were examined by western blotting at the indicated days post-infection. **f** The production of IFN-α in the serum was evaluated by ELISA analysis. * *p* < 0.05, ** *p* < 0.01, *** *p* < 0.001, compared to the group without fucoidan treatment. Data shown are representative of three independent experiments

After determination of the effect of fucoidan in suppressing HBV replication in vivo and in vitro, we next moved on to elucidate the underlying mechanism. It has been reported that activation of MAPK pathway could lead to the suppression of HBV replication [19]. We, therefore, sought to determine whether the MAPK members (i.e. ERK1/2, p38, and JNK) were activated in HepG2.2.15 cells by fucoidan treatment. The level of phosphorylated ERK was significantly increased after stimulation with 100 μg/ml of fucoidan, while the phosphorylation of p38 and JNK was comparable between the cell with or without fucoidan treatment (Fig. 4a).

To determine whether fucoidan has the capacity to regulate type I interferon response, we examined the effect of fucoidan on the activation of interferon regulatory factor 3 (IRF3) and IRF7 in HepG2.2.15 cells. The result showed that fucoidan significantly promoted the phosphorylation of IRF3 and IRF7 in the HBV-transfected cells (Fig. 4b). Also, fucoidan enhanced the production of IFN-α at both mRNA level and protein level (Fig. 4c and d). To support these findings, we next investigated whether fucoidan has the capacity to regulate type I interferon response in vivo. Western blotting showed more phosphorylated IRF7 and ERK in the liver of mice with fucoidan treatment than that of control group (Fig. 4e). Meanwhile, decreased level of IFN-α was detected in the serum samples from fucoidan-treated HBV-infected mice compared to those from control mice. (Fig. 4f). These results demonstrated that fucoidan selectively activated ERK pathway and subsequently enhanced type I interferon response in vitro and in vivo.

### Blockage of ERK activation abolished the inhibitory activity of fucoidan on HBV replication

**Fig. 5:**
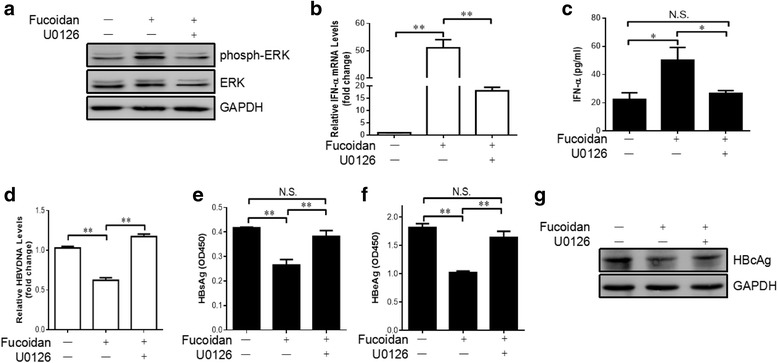
Fucoidan suppresses the replication of HBV and increases the production of type I IFN via activating the ERK pathway. HepG2.2.15 cells were left untreated or pretreated with U0126 (2 μM) for 2 h, followed by treatment with fucoidan (100 μg/ml) for 24 h. **a** Phospho-ERK and total ERK were detected by western blot. GAPDH was served as a loading control. **b** The mRNA level of IFN-α in cells was determined by quantitative RT-PCR. **c** The production of IFN-α in the culture supernatant was evaluated by ELISA analysis. **d** The HBV DNA replicative intermediates in the cells were detected by quantitative PCR. **e**, **f** The levels of HBsAg (**e**) and HBeAg (**f**) in the culture medium were measured by ELISA analysis. * *p* < 0.05, ** *p* < 0.01, N.S., not significant. **g** The expression of HBcAg in the cells was determined by western blot analysis. All the experiments were repeated at least three times independently

To validate the impact of ERK activation on fucoidan-mediated anti-viral effect, HepG2.2.15 cells were treated with U0126 to block ERK activation prior to incubation with 100 μg/ml of fucoidan. The result indicated that 2 μM of U0126 is sufficient to block ERK activation (Fig. 5a). After ERK inhibition, fucoidan-mediated enhancement of expression of IFN-α was significantly inhibited at either mRNA level or protein level (Fig. 5b and c). In the culture supernatants, the level of HBV-DNA (Fig. 5d) and secretion of HBsAg and HBeAg (Fig. 5e and f) were comparable between the control cells and fucoidan-treated cells with U0126 pretreatment. The data indicated that fucoidan-mediated inhibition of HBV replication was disturbed after pretreatment with ERK inhibitor. The western blotting analysis of the expression of HBcAg in the cells supported the above results (Fig. 5g). Together, these results strongly indicated that the fucoidan-mediated suppression of HBV was dependent on ERK activation.

## Discussion

Fucoidan, a natural sulfated polysaccharide that exists mainly in the cell wall matrix of various species of brown seaweed, has been shown to possess anti-cancer, anti-oxidant, anti-obesity and anti-diabetic effects [5, 20]. In this study, we demonstrated that fucoidan effectively inhibits HBV replication, and its mechanism partially occurs through the activation of ERK pathway and increased production of type I interferon.

It has been suggested that fucoidan has antivirus activity against many virus, such as HIV [12], herpes simplex virus (HSV) [13], influenza A virus [14], and newcastle disease virus [21]. Mori et al., previously found that fucoidan from *Cladosiphon okamuranus* has HCV replication suppressive effects in the cellular analysis [16]. After oral administration of fucoidan, HCV-infected patients showed a lower level of serum alanine aminotransferase, correlated with a significant decrease in HCV RNA. Therefore, we expected that fucoidan might have potentially beneficial effects in patients with chronic hepatitis B virus infection. To our knowledge, this study reported for the first time that fucoidan effectively inhibited HBV replication in vivo and in vitro. We found that treatment with fucoidan inhibited the total HBVRNA and the replication of HBV DNA replicative intermediates. Moreover, fucoidan treatment also reduced the amount of HBV core protein and the secretion of HBsAg and HBeAg. Given that the brown seaweeds containing fucoidan are widely consumed as part of the regular diet in East Asia (particularly China, Japan, and Korea), fucoidan is expected to have less drug-resistance compared to currently available anti-HBV agents. However, the mechanism of the effect of fucoidan on HBV gene expression should be elucidated with a variety of studies in vitro and in vivo, which remain need further investigation.

Activation of the MEK-ERK pathway has been reported to enhance the replication of viruses, such as HIV [22], influenza [23], and HSV [24]. By contrast, in the case of HBV, activation of MEK-ERK signaling led to the inhibition of HBV replication [19]. Several previous researches have pointed out that fucoidan could upregulate the phosphorylation of ERK in immune cells in response to diverse pro-inflammatory signals [25, 26]. Our data here demonstrated that fucoidan also increased the levels of phosphorylated ERK in hepatocytes and simultaneously suppressed HBV replication. When the specific inhibitors of ERK were added, both ERK phosphorylation and the anti-HBV activity of fucoidan were blocked. Taken together, the results revealed that fucoidan activated MEK-ERK signaling pathway contributes to the inhibition of HBV replication. It remains to be determined how fucoidan activates ERK in the hepatocyte.

In the early phase of HBV infection, type I IFN also controls viral infection by directly inducing antiviral infection in cells [2, 27]. IRF3 and IRF7, which are highly homologous, has been shown to play a role in the transcriptional activation of virus-inducible cellular genes, especially type I IFN gene expression [28]. Upon viral infection, IRF3 and IRF7 reside in the cytosol and undergoes serine phosphorylation in its C-terminal region, allowing their homo- or heterodimerization and nuclear translocation. The dimers then translocate to the nucleus and activate the transcription of type I IFN genes [28]. Fucoidan was thought to inhibit virus infection via blocking virus adsorption to the cell surface and subsequent virus entry [29]. Our current study provided the evidence that fucoidan enhanced the activation of IRF3 and IRF7 in the condition of HBV infection, which expands our understanding of the mechanism of fucoidan-mediated antiviral effect. Indeed, Wang et al., observed that virus infection of primary mouse embryo fibroblasts elicited ERK signaling, which was integrated into IRF3/7 activation and type I interferon induction [30]. Interestingly, the inhibitor of ERK U0126 attenuated fucoidan inducing IFN-α production, which suggests that fucoidan promotes type I interferon immune responses by activating ERK.

## Conclusion

We conclude that fucoidan inhibits HBV replication in vivo and in vitro. Our results demonstrated that fucoidan suppresses HBV gene expression by activating the ERK signaling pathway and subsequently enhancing the production of type I interferon. This new mechanism suggests another possible approach to inhibit HBV replication. Furthermore, either fucoidan alone or fucoidan combined with other established anti-HBV agents may serve as a new therapeutic strategy for HBV prevention and treatment.

## Abbreviations

ERK: Extracellular signal-regulated kinase
HBcAg: Hepatitis B core antigen
HBeAg: Hepatitis B e antigen
HBsAg: Hepatitis B surface antigen
HBV: Hepatitis B virus
HCV: Hepatitis C virus
HIV: Human immunodeficiency virus
HSV: Herpes simplex virus
IFN: Interferon
IRF: Interferon regulatory factor
MAPK: Mitogen-activated protein kinase
